# Evidence Map of Cupping Therapy

**DOI:** 10.3390/jcm10081750

**Published:** 2021-04-17

**Authors:** Tae Young Choi, Lin Ang, Boncho Ku, Ji Hee Jun, Myeong Soo Lee

**Affiliations:** 1 Clinical Research Division, Korea Institute of Oriental Medicine, Daejeon 34054, Korea; superoung@kiom.re.kr (T.Y.C.); anglin2808@kiom.re.kr (L.A.); zhixi04@kiom.re.kr (J.H.J.); 2 Korean Convergence Medicine, University of Science and Technology, 217, Gajeong-ro, Yuseong-gu, Daejeon 34113, Korea; 3 Clinical Research Coordinating Team, Korea Institute of Oriental Medicine, Daejeon 34054, Korea; secondmoon@kiom.re.kr

**Keywords:** evidence map, cupping therapy, systematic review, evidence synthesis

## Abstract

This study aimed to describe and assess the current evidence in systematic reviews on cupping therapy for various conditions. We searched PubMed, EMBASE, Cochrane Database of Systematic Reviews, China National Knowledge Infrastructure, and six Korean databases for systematic reviews of trials on cupping treatments for any condition published prior to March 2021. We used a bubble plot to graphically display the clinical topics, the number of articles, the number of participants in the total population, confidence, and effectiveness. Thirteen systematic reviews that met the inclusion criteria were included in the evidence map, and 16 bubbles were created. The findings from six reviews showed potential benefits of cupping for conditions such as low back pain, ankylosing spondylitis, knee osteoarthritis, neck pain, herpes zoster, migraine, plaque psoriasis, and chronic urticaria. Cupping has been applied in a variety of clinical areas, and systematic reviews in a few of these areas have demonstrated statistically significant benefits. The evidence map provides a visual overview of cupping research volume and findings. Evidence mapping can facilitate the transfer of knowledge from researchers to policymakers and promote research on musculoskeletal pain (such as low back pain, neck pain, and knee osteoarthritis) and skin disease (plaque psoriasis).

## 1. Introduction

Cupping has been used with traditional and alternative medicine to treat a variety of conditions in China, Korea, and East Asia. In Taiwan, 12.8% of participants in a previous study reported undergoing cupping therapies in the past year [1]. Cupping has also recently gained popularity in Western countries such as Europe, the United States, and Arabia [2]. Although it is a timeworn technique, it is still being used in the treatment of various medical conditions [3], especially those involving pain, such as low back pain [4], neck pain [5], and knee osteoarthritis [6].

Cupping, whether it is performed dry or wet, is a technique in which cups made of plastic, bamboo, or glass cups are used in suctioning the skin over acupuncture points, painful areas, or reflex zones [7]. For dry cupping, the cup creates a mild vacuum on the skin to aggravate the subcutaneous tissues without blood being drawn. For wet cupping, the cup suctions the lacerated skin to draw blood from the dermal microcirculation. Cupping has also been used to improve subcutaneous blood flow to skin and muscles and to stimulate the autonomic nervous system [7,8]. Besides, cupping is also used for draining excess fluids and toxins, loosening adhesions, and lifting connective tissues [8]. Correspondingly, in the theory of traditional medicine, cupping promotes the circulation of Qi and blood of the treatment area to alleviate pain and tension caused by stagnation, and expels the pathogenic factors, eventually leading to the restoration of physiological harmony and balance.

The usage of cupping therapies to improve health outcomes has been increasing continuously, resulting in cupping being the subject of investigation in many primary studies and systematic reviews (SRs). However, previous research has covered a variety of conditions, populations, and settings. Several SRs have investigated the therapeutic effect of cupping therapy for low back pain [9], neck pain [10], and hypertension [11]. An overview of SRs identified 8 reviews published in 2015 and concluded that cupping may aid in pain-related conditions, acne, and facial paralysis [12]. However, previous reviews are outdated and reported in insufficient detail pertaining to the included studies. Moreover, they did not use graphical representations, which facilitate the interpretation of results.

Evidence maps are new synthesis tools that involve searching systematically and characterizing existing research on a topic of interest for the identification of knowledge gaps and future research needs [13]. We conducted an evidence mapping analysis to determine the distribution of evidence available regarding the indication of cupping for various conditions as well as different forms of cupping therapy; this form of systematic literature synthesis visualized the volume and the topics of research.

This evidence map study aimed to present a visual overview of the distribution of evidence available on cupping therapy for various conditions, along with a concomitant narrative that will aid stakeholders in interpreting the state of the current evidence for informed policy and clinical decision making.

## 2. Methods

### 2.1. Study Design

There are no official standardized methods for evidence mapping [14]. The methods used in this study were based on those reported by Hempel et al., in the “Evidence Map of Acupuncture” [15].

### 2.2. Electronic Searches and Search Strategy

The following electronic databases were systematically searched for in literature published from their dates of inception to 16 March 2021: PubMed, EMBASE, the Cochrane Database of Systematic Reviews (CDSR), one Chinese database (the China National Knowledge Infrastructure (CNKI)), and six Korean medical databases (Research Information Service System (RISS), DBpia, Korea Med, Oriental Medicine Advanced Search Integrated System (OASIS), Korean Studies Information Services System (KISS), and Korean Medical Database (KM base)). In addition, the reference lists of the potentially eligible articles were searched manually to further identify additional eligible papers.

The search terms used were “systematic review” or “meta-analysis” and “cupping therapy”, and the database-specific filters were used to search for SRs without language restrictions (Supplement 1).

### 2.3. Inclusion Criteria

#### 2.3.1. Design

Only SRs focusing on cupping therapy, which compiled primary research studies of any clinical indications, were included. We defined SRs as self-identified review articles that included the phrase “systematic review” in the title or the main text and/or review articles that reported the sources systematically searched for in the identified studies. SRs are studies in which the findings of studies are systematically selected from the medical literature and are summarized for each type of intervention.

#### 2.3.2. Population

Individuals with any conditions were included.

#### 2.3.3. Intervention

SRs of the effectiveness of cupping for any clinical indication were eligible for inclusion. All trials were included regardless of the type of cupping and control intervention. Trials that used cupping as an adjunctive treatment were also included.

#### 2.3.4. Outcomes

SRs that report patient health outcomes were included and those of provider outcomes, study designs, or intervention features unrelated to patient health outcomes were excluded.

#### 2.3.5. Timing

SRs that summarize the assessments of interventions of any duration with any follow-up period were included.

#### 2.3.6. Systematic Review Selection

Formal SR articles with and without a meta-analysis related to any type of cupping therapy for any type of medical condition were eligible for inclusion. Articles that were not performed using systematic methods such as reviews, comments, and overviews, were excluded. Two reviewers (TYC and JHJ) independently screened all the titles and abstracts and read the full text of articles to exclude irrelevant SRs. Disagreements were resolved through discussion and consensus, and an additional reviewer (MSL) was consulted. The originals and updates of SRs by the same author group were always available, but only the most recent version was considered. In the case of multiple publications, they were considered as one review, and data were extracted from all the available publications. If multiple reviews of similar clinical topics were identified, the most pertinent and best-performed SR was used for the inclusion of evidence maps selected based on the results of the AMSTAR 2 assessment.

### 2.4. Methodological Quality Assessment

The Assessing the Methodological Quality of Systematic Reviews (AMSTAR)-2 tool was used to critically appraise the quality of reporting for each included SR. A validated 16-item instrument was also used for the appraisal of the SRs based on critical flow and bias using ratings of “yes”, “partial yes”, or “no” [16]. The overall confidence in the results of the SRs was then judged using the following four categories: “high” for none or one noncritical weakness, “moderate” for more than one noncritical weakness, “low” for one critical flaw with or without noncritical weaknesses, and “critically low” for more than one critical flaw with or without noncritical weaknesses.

### 2.5. Data Extraction

Information regarding the population, intervention, comparison and outcomes (PICO) process, the numbers of randomized controlled trials (RCTs) included in each SR, summary effect estimates for the main outcomes, overall risk of bias of the included RCTs, publication bias, and conclusions as quoted from the original article, were extracted. The information for each intervention was extracted separately if the SR was designed as an overview or an umbrella review. All articles were read by two independent reviewers (TYC and JHJ), and the data were extracted from the articles according to the predefined criteria for the data extraction and methodological quality assessments. Disagreements were resolved by consensus, and when necessary, an additional reviewer (MSL) participated in the discussion.

### 2.6. Evidence Map Presentation and Domains

We used topics of investigation of the included SRs to categorize them. The results of evidence mapping were presented using characteristic tables of included SRs and a graphical display of bubble plots. Each bubble in the chart represents the included evidence for clinical topics as assessed by the SR investigating the effectiveness of cupping.

The outcomes, populations, or clinical indications were focused in the reviews. The SRs were grouped into clinical topics by one reviewer, and the grouping results were discussed and reviewed by the team. The decisions to not combine potentially related topic areas were due to the lack of overlapping between studies included in the reviews and differences in the reported outcomes or the review’s conclusion. When SRs contained various diseases, each disease was analyzed separately and used multiple times in the bubble plot. The evidence map used R software (version 3.5.0; R Foundation for Statistical Computing, Vienna, Austria).

This chart displays the information in four dimensions:

(1) *X*-axis: effect estimate

The clinical effectiveness of cupping therapy was evaluated based on the effect estimates and the overall risk of bias reported in the selected SRs. Clinical effectiveness was classified as “effective” when the effect estimates were significantly positive with a low overall risk of bias, “potentially effective” when the effect estimates were significantly positive with a high overall risk of bias, or “unclear” when the effect estimates were negative or the overall risk of bias was unclear.

(2) *Y*-axis: number of articles

The literature size was defined as the number of RCTs in the selected SRs on the topic. Although reviews often differ in their inclusion criteria, all reviews are likely to include studies of well-established research design, such as an RCT, and provides a broad estimate of the research volume.

(3) Bubble size: number of participants in the total population

The size of each SR bubble was proportional to the number of participants in the total sample size for the effects of cupping.

(4) Color: strength of the findings

Confidence was determined based on the results of the AMSTAR 2 assessment and was classified into the following four categories: yellow circles correspond to “high confidence”; green circles correspond to “moderate confidence”; blue circles correspond to “low confidence”, or red circles corresponded to “critically low confidence”.

### 2.7. Narrative Synthesis

To provide more details from the included SRs, a concomitant narrative synthesis was expanded upon the visual evidence map. Such details included descriptions of the findings, the features of cupping therapy, and the types of diseases.

## 3. Results

### 3.1. Description of Included SRs

#### 3.1.1. Selection Diagram

**Figure 1:**
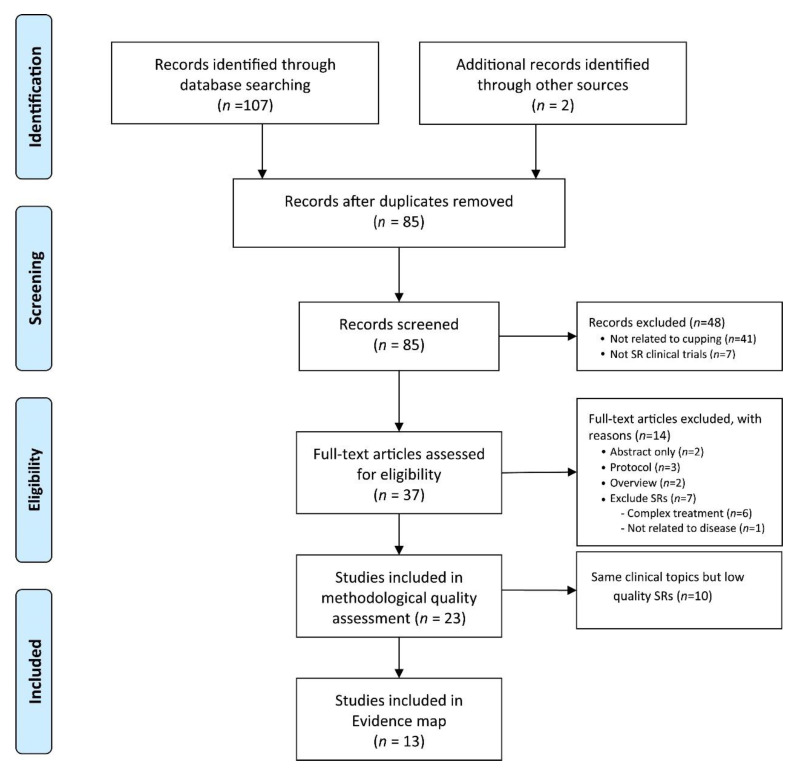
PRISMA diagram for the included studies. SR: systematic review.

The electronic database search identified 107 potentially relevant studies. After duplicate publications were removed, the titles and abstracts of 85 papers were screened. The full texts of 37 articles were reviewed for eligibility and 23 SRs were included in the review. According to the results of the AMSTAR 2 assessment, only the best performed SRs were selected for further inclusion (Figure 1). Finally, 13 SRs [9,10,11,17,18,19,20,21,22,23,24,25] that met the inclusion criteria were included in the evidence map, and 16 bubbles were created. The studies selected were conducted in 3 countries: Brazil (*n* = 1), China (*n* = 9), and Korea (*n* = 3). The information of the excluded 10 SRs information is presented in Supplements 2 and 3.

#### 3.1.2. Included Diseases

The effectiveness, literature size, and confidence level for cupping therapy in the included SRs were identified and evaluated. A few SRs covered several types of diseases, which were assessed separately in our evaluation. The medical conditions studied in the RCTs of the included SRs were back pain (*n* = 7), ankylosing spondylitis (*n* = 2), knee osteoarthritis (*n* = 3), neck pain (*n* = 4), cervical spondylosis (*n* = 1), lateral femoral cutaneous neuritis (*n* = 1), scapulohumeral periarthritis (*n* = 1), herpes zoster (*n* = 4), facial paralysis (*n* = 1), acne (*n* = 1), cervical spondylosis (*n* = 1), stroke rehabilitation (*n* = 1), hypertension (*n* = 2), migraine (*n* = 1), plaque psoriasis (*n* = 2), chronic urticarial (*n* = 1), and obesity (*n* = 1).

#### 3.1.3. Intervention Components Described

Given that there are many types of cupping therapies, accurate and detailed reporting about these interventions is necessary to understand which therapies are included and synthesized in the SRs. A description of the cupping style was included in all the SRs. Only two SRs focused on one type of cupping, which is wet cupping [11] and moving cupping [17], whereas the other SRs included all types of cupping. Cupping has been applied in a variety of clinical areas, and systematic reviews in a few of these areas have demonstrated statistically significant benefits (Table 1).

### 3.2. Quality of the Included Systematic Reviews

Regarding the quality assessments of the overall confidence level for each SR, most reviews showed a moderate to critically low quality. All the reviews included comprehensive searches, interpreted the results based on the risk of bias assessment results, and reported any conflicts of interest. No reviews mentioned the study lists that were excluded or provide a satisfactory explanation for the heterogeneity in their results. The overall confidence was rated as “moderate” for six SRs [10,17,18,19,22,25], “low” for six SRs [9,11,20,21,24,26], and as “critically low” for one SR [23] (Table 2).

### 3.3. Effectiveness

For the overlapping diseases, overall confidence was considered. The conclusions were reflected in the individual SRs and confirmed through an internal review.

#### 3.3.1. Evidence of a Positive Effect

The effects of cupping, as indicated by statistically significant pooled treatment effects in SRs, were determined based on a substantial number of research studies that included findings on low back pain.

#### 3.3.2. Evidence of a Potentially Positive Effect

Promising effects of cupping, as indicated by statistically significant pooled treatment effects in SRs, were determined based on a substantial number of research studies on conditions including ankylosing spondylitis, knee osteoarthritis, neck pain, herpes zoster, migraine, plaque psoriasis, and chronic urticaria.

#### 3.3.3. Evidence of Unclear

The map includes a small number of SRs that provided evidence of the potential lack of effectiveness of cupping in treating clinical conditions, e.g., cervical spondylosis, lateral femoral cutaneous neuritis, scapulohumeral periarthritis, facial paralysis, acne, stroke rehabilitation, hypertension, and obesity, based on more than one included study.

### 3.4. Evidence Map

**Figure 2:**
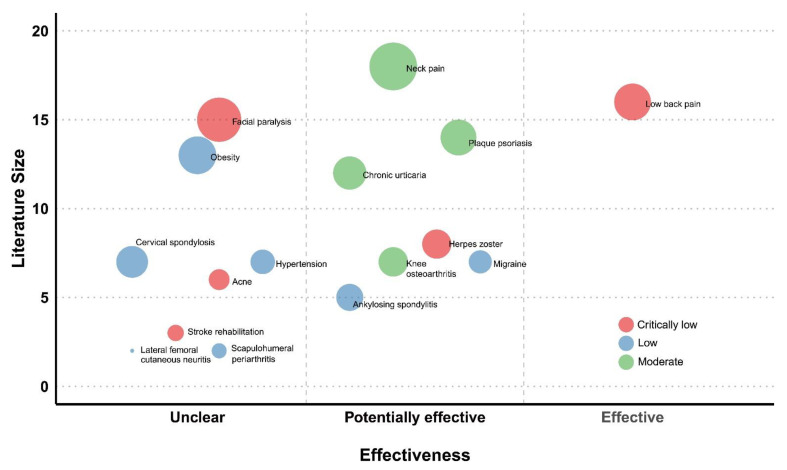
Evidence map of cupping therapy.

Figure 2 visualized the results of the evidence mapping. The evidence map shows the evidence of each included SR in the form of bubbles. As described in the prior Materials and Methods section, each bubble label represents the medical condition investigated in that review. The bubble size indicates the total sample size of a particular medical condition studied for the effectiveness of cupping as similar primary studies could be included in multiple SRs. Each bubble was plotted according to the strength of the findings for cupping (color), the effect of cupping (*x*-axis), and the number of primary studies (*y*-axis). The details of the included SRs were provided in Table 1 and Table 2.

All included SRs concluded that cupping did not improve the outcomes of interest; however, the number of existing studies was small in all of the identified topic areas. The evidence mapping results showed that only a restricted number of cupping therapies were assessed with inconclusive clinical evidence, indicating the necessity of additional primary research in this area (Figure 2).

## 4. Discussion

The evidence map visualizations show the presence and absence of evidence for cupping therapy based on 13 published SRs and identified the evidence gaps relating to the details of the cupping therapy in particular. The studies included in our evidence map provide a very extensive and broad overview of the evidence on cupping therapy published between 2010 and 2020. Our evidence map also highlights the areas where meta-analyses have reported positive or unclear results, in the meantime showing the research concentration and volume. This evidence mapping process shows a range of evidence on cupping for any condition. Cupping has been clinically utilized and evaluated for a wide range of clinical conditions. Several identified SRs have addressed a very broad topic such as low back pain even though they included a large number of RCTs. Additionally, evidence on the role of cupping for specific clinical conditions is also very limited due to the small number of published studies.

Even though evidence maps can provide only a broad overview of research areas, our review concluded that more rigorous research on the clinical effectiveness of cupping is needed across various clinical topic areas. The duration and frequency of the cupping interventions in the cited studies have yet to be evaluated and need to be studied further. Furthermore, such inconclusive evidence also warrants more research.

The findings of seven reviews showed evidence of potential benefits of cupping for conditions including neck pain, knee osteoarthritis, plaque psoriasis, chronic urticarial, ankylosing spondylitis, herpes zoster, and migraine. Many studies have provided evidence concerning the effectiveness of cupping in treating certain clinical indications. Cupping therapy is a traditional method and is currently used in the treatment of a broad range of medical and health conditions. Nevertheless, the mechanism of action of cupping remains unclear and is yet to be fully understood [27].

Evidence maps are one of many tools and information sources to support evidence. It is similar to systematic review methods that are reproducible and transparent. However, while SRs target specific research questions, evidence mapping focuses on the nature, volume, and characteristics of the research to identify, describe and summarize what is known [28]. The creation and publication of evidence maps consist of graphically representing the highest quality evidence found, analyzing and categorizing it, and linking it with the bibliographic records and full texts of the studies to facilitate access to information for everyone interested.

This evidence map has described the research focus, which was reported in the existing SRs, and identified the gaps in evidence to pinpoint areas that should be prioritized in future research. However, this evidence map is unable to answer more refined questions, such as the most adequate cupping therapy for specific applications and the differences between health services. To further advance our evidence-based knowledge of cupping, we should collect more data on the effectiveness of cupping across and within each clinical condition and patient population through meta-analyses across primary studies. Additionally, the large number of topic areas that were classified as having unclear evidence warrants additional primary studies. More studies have been published in some of the topic areas included in the unclear evidence category, and the currently available SRs need to be updated. As there might be other efficient ways of drawing evidence maps, further research should also include developing evidence maps of other research designs.

In conclusion, cupping has been applied in a variety of clinical areas, and for a few of these, SRs have demonstrated statistically significant results. This evidence map provides a very broad overview of the evidence base, indicating the areas in which research has been conducted. The evidence map provides a visual overview of the cupping research volume and content. Evidence mapping can facilitate the transfer of knowledge from researchers to policymakers and promote research on musculoskeletal pain (as low back pain, neck pain, and knee osteoarthritis) and skin disease (plaque psoriasis).

## Figures and Tables

**Table 1:** Summary of the included systematic reviews of cupping therapy.

First Author (year) (Ref) Country	Condition Search Date No. of Primary Studies	Cupping Therapy	Comparator	Outcome	Overall Risk of Bias	Effect Estimates for Main Outcomes (Meta-Analysis)	Conclusion (Quoted from the Original Paper)	Overall Confidence
Moura (2018) [9] Brazil	Chronic back pain May 2018 16 RCTs	All types	-Sham -Waiting list -WM -None	Pain	High	MD −1.59 (−2.07, −1.10), *p* = 0.001	…has shown positive results …	Effective
Kim (2018) [10] Korea	Neck pain Jan 2018 18 RCTs	All types	-Usual care -AT -Waiting list -No treatment	(1)Pain (2)Function	High	vs. no treatment (1) MD −2.42 (−3.98, −0.86), *p* < 0.00001 (2) MD −4.34 (−6.77, −1.19), *p* = 0.0005 vs. active control (1) MD −0.89 (−1.42, −0.37), *p* = 0.0009 (2) MD −4.36 (−8.67, −0.04), *p* = 0.05	… reduce neck pain...	Potentially effective
Lu (2018) [11] China	Hypertension May 2018 7 RCTs	Wet cupping	-WM -AT	(1) SBP (2) DBP (3) Antihypertensive effect (4) Effective rate	High	(1) MD −2.24 (−9.13, 4.65), *p* = 0.52 (2) MD −2.11 (−8.85, 4.64), *p* = 0.54 (3) RR 1.09 (0.99, 1.20), *p* = 0.07 (4) RR 1.22 (1.05, 1.40), *p* = 0.007	… no firm conclusions …	Unclear
Ma (2018) [18] China	Ankylosing spondylitis Dec 2017 5 RCTs	All types	-Sham/placebo -WM	(1) BASFI (2) BASDAI (3) ESR	High	(1) MD −16.63 (−17.75, −15.51), *p* < 0.00001 (2) MD −9.93 (−10.34, −9.52), *p* < 0.00001 (3) MD −3.96 (−4.69, −3.23), *p* < 0.00001	…weak evidence …	Potentially effective
Li (2017) [19] China	Knee osteoarthritis Jan 2017 7 RCTs	All types	-Sham/placebo -WM	WOMAC (1) Pain (2) Stiffness (3) Physical function	High	vs. WM (1) MD −1.01 (−1.61, −0.41), *p* < 0.01 (2) MD −0.81 (−1.14, −0.48), *p* < 0.01 (3) MD −5.53 (−8.58, −2.47), *p* < 0.01	…weak evidence … cupping therapy…	Potentially effective
Zhang (2017) [20] China	Several conditions Mar 2017 23 RCT (Cervical spondylosis, 7 RCTs; lateral femoral cutaneous neuritis, 2 RCTs; scapulohumeral periarthritis, 2 RCTs; Others, 12 RCTs)	All types	AT	Effective rate	High	Cervical spondylosis RR 1.13 (1.01, 1.26), *p* = 0.04 Lateral femoral cutaneous neuritis RR 1.10 (1.00, 1.22), *p* = 0.71 Scapulohumeral periarthritis RR 1.31 (1.15, 1.51), *p* = 0.84	Cupping …safe… relieving pain.	Unclear
Cao (2010) [21] China	Herpes zoster Feb 2009 8 RCTs	Wet cupping	-No treatment -Placebo -WM	Effective rate	High	vs. WM RR 1.15 (1.91, 3.24), *p* = 0.0.005	…appears to be effective…	Potentially effective
Cao (2012) [22] China	Several conditions Dec 2010 135 RCTs Herpes zoster (15 RCTs) Facial paralysis (15 RCTs) Acne (6 RCTs) Cervical spondylosis (6 RCTs) Other conditions (93 RCTs)	All types	-WM -AT	Effective rate	High	vs. WM Herpes zoster RR 2.07 (1.77, 2.43), *p* < 0.00001 Facial paralysis RR 1.49 (1.35, 1.65), *p* < 0.00001 Acne RR 2.14 (1.40, 2.65), *p* = 0.0003 Cervical spondylosis RR 2.07 (1.77, 2.43), *p* < 0.00001	No confirm conclusion…	Unclear
Lee (2010a) [23] Korea	Stroke rehabilitation Mar 2010 5 studies (3 RCTs, 2 UOS)	All types	-AT	(1) Effective rate (2) VAS	High	(1) *p* < 0.05 (2) *p* = 0.004	Insufficient…	Unclear
Seo (2018) [24] Korea	Migraine Sep 2016 7 RCTs	All types	-WM -AT	(1) Effective rate (2) VAS	High	vs. WM (1) RR 1.22 (1.08, 1.37), *p* = 0.001 (2) MD −3.29 (−8.22, 1.64), *p* = 0.19 Cupping + AT vs. AT (1) RR 1.05 (0.99, 1.12), *p* = 0.13	…improves…effect of migraine …	Potentially effective
Xing (2020) [17] China	Plaque psoriasis Mar 2020 16 RCTs	Moving cupping	-Oral Chinese medicine -Placebo -WM	(1) Recovery rate (2) Recurrence rate (3) VAS	High	(1) SMD −1.22 (−1.58, −0.85), *p* < 0.00001 (2) RR 0.33 (0.16, 0.68), *p* = 0.003 (3) WMD −0.27 (–0.71, 0.17), *p* = 0.22	…could be an effective…	Potentially effective
Xiao (2020) [25] China	Chronic urticaria May 2019 12 RCTs	All types	-AT -WM	(1) Effective rate (2) Recurrence rate	High	Wet cupping vs. WM (1) RR 1.10 (0.97, 1.25), *p* = 0.14 (2) RR 0.56 (0.23, 1.36), *p* = 0.20 Cupping + WM vs. WM (1) RR 1.18(1.01, 1.39), *p* = 0.03 (2) RR 0.52(0.32, 0.84), *p* = 0.007	… it may enhance the efficacy	Potentially effective
Yang (2020) [26] China	Obesity June 2019 13 RCTs	All types	-AT -Cupping	(1) Effective rate (2) Weight (3) BMI (4) Waist circumference	High	AT + cupping vs. AT (1) OR 2.28 (1.56, 3.32), *p* < 0.0001 (2) SMD −0.21 (−0.36, −0.06), *p* = 0.007 (3) SMD −0.69 (−0.85, −0.54), *p* < 0.00001 (4) SMD −0.46 (−0.75, −0.17), *p* = 0.002 AT + cupping vs. cupping (1) OR 8.79 (4.20, 18.40), *p* < 0.0001 (2) SMD −0.54 (−0.79, −0.29), *p* < 0.001 (3) SMD −0.42 (−0.67, −0.17), *p* = 0.001 (4) SMD −0.46 (−0.75, −0.17), *p* = 0.002	Insufficient…	Unclear

AT: acupuncture; BASFI: Bath Ankylosing Spondylitis Functional Index; BASDAI: Bath Ankylosing Spondylitis Disease Activity Index; BMI: body mass index; DBP: diastolic blood pressure; ESR: Erythrocyte sedimentation rate; LAI: Lequesne Algofunctional Index; NR: Not reported; NRS: numerical rating scale; OR: odd ratio; PHN: Postherpetic neuralgia; RR: risk ratio; RCT: randomized controlled trials; SBP: systolic blood pressure;; UOS: uncontrolled observational studies; VAS: Visual analog scale; WM: Western medicine; WMD: weighted mean difference; WOMAC: Western Ontario and McMaster Universities Osteoarthritis Index.

**Table 2:** Quality assessment of all included systematic reviews using AMSTAR 2.

Study ID	1	2	3	4	5	6	7	8	9	10	11	12	13	14	15	16	Rating Overall Confidence *
Moura (2018) [9]	Yes	No	Yes	Yes	No	No	No	Yes	Yes	Yes	Yes	Yes	Yes	No	No	No	Critically low
Kim (2018) [10]	Yes	Yes	Yes	Yes	Yes	Yes	No	Yes	Yes	Yes	Yes	Yes	Yes	No	No	Yes	Moderate
Lu (2018) [11]	Yes	No	Yes	Yes	Yes	Yes	No	Yes	Yes	No	Yes	Yes	Yes	No	No	Yes	Low
Ma (2018) [18]	Yes	Yes	Yes	Yes	Yes	No	No	Yes	Yes	Yes	Yes	Yes	Yes	No	No	Yes	Low
Li (2017) [19]	Yes	Yes	Yes	Yes	Yes	Yes	No	Yes	Yes	Yes	Yes	Yes	Yes	No	No	Yes	Moderate
Zhang (2017) [20]	Yes	Yes	Yes	Yes	Yes	Yes	No	Yes	Yes	Yes	Yes	Yes	Yes	No	No	Yes	Low
Cao (2010) [21]	No	No	Yes	Yes	No	Yes	No	Yes	Yes	Yes	Yes	No	Yes	No	Yes	Yes	Critically low
Cao (2012) [22]	No	No	Yes	Yes	Yes	Yes	No	Yes	Yes	Yes	Yes	No	Yes	No	Yes	Yes	Critically low
Lee (2010a) [23]	Yes	No	No	Yes	No	Yes	No	Yes	Yes	Yes	No-MA	No-MA	Yes	No-MA	No-MA	Yes	Critically low
Seo (2018) [24]	Yes	Yes	Yes	Yes	Yes	No	No	Yes	Yes	No	Yes	No	Yes	Yes	No	No	Low
Xing (2020) [17]	Yes	Yes	Yes	Yes	Yes	Yes	No	Yes	Yes	Yes	Yes	Yes	Yes	Yes	Yes	Yes	Moderate
Xiao (2020) [25]	Yes	No	Yes	Yes	Yes	Yes	No	Yes	Yes	Yes	Yes	Yes	Yes	Yes	No	Yes	Moderate
Yang (2020) [26]	Yes	No	Yes	No	Yes	Yes	No	Yes	Yes	Yes	Yes	Yes	Yes	No	Yes	No	Low

AMSTAR 2: A MeaSurement Tool to Assess systematic Reviews 2; No-MA: No meta-analysis conducted. 1. components of PICO/2. established prior to the conduct of the review/3. explain their selection of the study designs/4. comprehensive search/5. duplicate selection/6. duplicate extraction/7. list of excluded studies and justify the exclusions/8. describe the included studies in adequate detail/9. use a satisfactory technique for assessing the risk of bias (RoB)/10. report on the sources of funding/11. use appropriate methods for statistical combination of results/12. assess the potential impact of RoB in individual studies on the results/13. account for RoB in individual studies when interpreting/discussing the results of the review?/14. Did the review authors provide a satisfactory explanation for, and discussion of, any heterogeneity observed in the results of the review/15. Publication bias assessed/16. Include conflict of interest * AMSTAR2 was used to critically appraise the reporting quality of each included SR. The overall confidence of each SR was graded as “high”, “moderate”, “low” or “critically low”.

## Data Availability

Data sharing not applicable.

## Supplementary Materials

The following are available online at https://www.mdpi.com/article/10.3390/jcm10081750/s1, Supplement 1: Search strategy. Supplement 2. CNKI Chinese database. Supplement 3. Korea Med Korean database.
