# Supplementary material for: Evidence Map of Cupping Therapy
Source: J Clin Med. 2021 Apr 17;10(8):1750. doi: 10.3390/jcm10081750 (PMC8073851; doi:10.3390/jcm10081750)
Supplement: Supplementary file 1 [file jcm-10-01750-s001.zip › jcm-1162501-supplementary.pdf]

Supplement 1. Search strategy

1. PubMed database

1. Cupping [Mesh]
2. Cupping [Title/Abstract]
3. cups [Title/Abstract]
4. “Meta-Analysis” [Publication Type]
5. “meta-analysis” [Title/Abstract]
6. “systematic review” [Title/Abstract]
7. OR/1-3
8. OR/4-6
9. 7 AND 8

2. CNKI Chinese database

1. 拔罐
2. 火罐
3. 走罐
4. 闪罐
5. 刺血拔罐
6. 放血拔罐
7. Wet cupping
8. Bloodletting and cupping
9. Cupping

10. 系统评价

11. Meta分析

12. 荟萃分析

13. OR/ 1-9

14. OR/10-12

15. 13 AND 14

3. Korea Med Korean database

부항 AND (체계적 문헌고찰 OR 메타분석)

Supplement 2. Summary of the excluded systematic reviews of cupping therapy.

First author (year) Country	Condition Search date No. of primary studies	Cupping therapy	Comparator	Outcome	Overall risk of bias	Effect estimates for main outcomes (meta-analysis)	Conclusion (quote from the original paper)	Overall confidence*
Wang (2017) [1] China	Low back pain<br>Aug 2016<br>6 RCTs	All types	-WM<br>-Usual care	1)VAS<br>2)ODI<br>3)MPPI	High	1) SMD -0.73 (-1.42, -0.04), P=0.04<br>2) SMD -3.64 (-5.85, -1.42), P=0.001<br>3) SMD -6.12 (-14.54, 2.31), P=0.15)	…can significantly decrease the VAS and ODI…	Potentially effective
Huang (2013) [2] China	Low back pain<br>n.r. 2013<br>7 studies<br>(1 RCT, 6 non-RCTs)	All types	-Waiting list<br>-WM	Effective rate	High	P >0 .05	…is promising for pain control and improves QoL and safe.	Potentially effective
Azizkhani (2018) [3] Iran	Non-specific neck pain<br>March 2017<br>10 RCTs	All types	-Waiting list<br>-Usual care	1)VAS<br>2)NRS<br>3)SF36	High	1) SMD -0.84 (-1.22, -0.46), P=0.065<br>2) SMD -0.60 (-0.86, -0.35), P=0.308)<br>3) SMD -0.56 (-0.20, -0.92), P=0.083	… improve…neck pain	Potentially effective
Cao (2014) [4] China	Several conditions<br>Dec 2013<br>16 RCTs<br>Herpes zoster (2 RCTs)<br>Neck pain (4 RCTs)<br>Low back pain (2 RCTs)<br>Osteoarthritis (2 RCTs)<br>Other conditions (6 RCTs)	All types	-No treatment<br>-Usual care	1)VAS<br>2)NRS	High	1) (vs. waiting list)<br>MD -1.85 (-2.66, -1.04), P<0.00001<br>2) (vs. heat therapy)<br>MD -2.05 (-2.93, -1.17), P<0.00001)	… short-term effect on reducing pain…	Potentially effective
Kim (2011) [5] Korea	Several conditions<br>Jan 2009<br>7 RCTs<br>Low back pain (2 RCT)<br>Other conditions (5 RCT)	All types	-Usual care<br>-Analgesia<br>-Heat pad	Pain intensity	High	P < 0.01	More rigorous studies are required …	Potentially effective
Kwon (2007) [6] Korea	Several conditions<br>Jan 2007<br>5 studies<br>(2 RCT, 3 CCTs)<br>Low back pain (1 RCT)<br>Ankle sprain (1 RCT)	Wet cupping	AT	Pain rating scale	High	WMD 10.45 (1.11, 19.80), P=0.03	…. low back pain is effective….	Potentially effective
Wood (2020) [7] Australia	Several conditions<br>April 2018<br>21 RCTs<br>Neck pain (7 RCTs)<br>Low back pain (3 RCTs)<br>Other conditions (11 RCTs)	Dry cupping	-WM<br>-Sham<br>- No treatment	1)VAS<br>2)NDI<br>3)Pressure pain threshold	High	Neck pain<br>1) MD -21.67 (-36.55, -6.80), P=0.004<br>2) SMD -4.65 (-6.44, -2.85), P<0.0001<br>3) MD -0.40 (-0.69, -0.11), P=0.006<br><br>Low back pain<br>MD -19.38 (-28.09, -10.66), P<0.0001	…effective for reducing pain … definitive conclusions regarding the effectiveness and safety of cupping for musculoskeletal pain … unable to be made ...	Unclear
Al Bedah (2016) [8] Saudi Arabia	Several conditions<br>Feb 2016<br>14 RCTs<br>Low back pain (3 RCTs)<br>Neck pain (2 RCTs)<br>Other conditions (9 RCTs)	Wet cupping	-No treatment<br>-Active controls	Pain	High	P < 0.01 or P<0.05	… promising evidence …wet cupping in musculoskeletal pain…	Potentially effective
Lee (2010)b [9] Korea	Hypertension<br>June 2009<br>2 studies<br>(1 RCT, 1 UOS)	All types	WM	Changes in cerebral vascular function	High	P<0.05	…not significantly convincing …	Unclear
Li (2020) [10] China	Psoriasis vulgaris<br><br>Dec 2019<br>14 RCTs	All types	-Oral Chinese medicine	Effective rate	High	RR 3.32 (2.32, 4.75), P<0.00001	… showed that the combined with cupping therapy had obvious advantages …	Potentially effective

AT: acupuncture; NDI: Neck Disability Index; NRS: numerical rating scale; MPPI: McGill present pain index; ODI: Oswesty pain disability index; QoL: quality of life; RR: risk ratio; RCT: randomized controlled trials; SF36: 36-item short form survey; UOS: uncontrolled observational studies; VAS: Visual analog scale; WM: Western medicine; WMD: weighted mean difference.

Supplement 3. Quality assessment of all excluded systematic reviews using AMSTAR 2.

Study ID	1	2	3	4	5	6	7	8	9	10	11	12	13	14	15	16	Rating overall confidence*
Wang (2017)[1]	Yes	No	Yes	Yes	Yes	Yes	No	Yes	Yes	Yes	Yes	Yes	Yes	No	No	Yes	Low
Huang (2013)[2]	Yes	No	Yes	No	No	No	No	Yes	No	No	No-MA	No-MA	Yes	No-MA	No-MA	Yes	Critically low
Azizkhani (2018)[3]	Yes	No	No	Yes	No	Yes	No	Yes	Yes	No	Yes	Yes	Yes	No	No	No	Low
Cao (2014)[4]	Yes	Yes	Yes	Yes	Yes	Yes	No	Yes	Yes	Yes	Yes	Yes	Yes	No	Yes	Yes	Moderate
Kim (2011)[5]	No	No	Yes	Yes	Yes	Yes	No	Yes	Yes	Yes	No-MA	No-MA	Yes	No-MA	No-MA	No	Critically low
Kwon (2007)[6]	No	No	Yes	Yes	No	Yes	No	Yes	Yes	No	Yes	Yes	Yes	No	No	No	Critically low
Wood (2020)[7]	Yes	Yes	Yes	Yes	Yes	Yes	No	Yes	Yes	No	Yes	Yes	Yes	No	Yes	Yes	Moderate
Al Bedah (2016)[8]	Yes	No	Yes	Yes	Yes	Yes	No	Yes	Yes	No	No-MA	No-MA	Yes	No-MA	No-MA	Yes	Critically low
Lee (2010)b[9]	No	No	No	Yes	Yes	Yes	No	Yes	Yes	Yes	No-MA	No-MA	Yes	No-MA	No-MA	Yes	Critically low
Li (2020)[10]	Yes	No	Yes	Yes	Yes	Yes	No	Yes	Yes	Yes	Yes	Yes	Yes	No	Yes	No	Low

AMSTAR 2: A MeaSurement Tool to Assess systematic Reviews 2; No-MA: No meta-analysis conducted. 1. components of PICO/ 2. established prior to the conduct of the review/ 3. explain their selection of the study designs/ 4. comprehensive search/ 5. duplicate selection/ 6. duplicate extraction/ 7. list of excluded studies and justify the exclusions/ 8. describe the included studies in adequate detail/ 9. use a satisfactory technique for assessing the risk of bias (RoB)/ 10. report on the sources of funding/ 11. use appropriate methods for statistical combination of results/ 12. assess the potential impact of RoB in individual studies on the results/ 13. account for RoB in individual studies when interpreting/ discussing the results of the review?/ 14. Did the review authors provide a satisfactory explanation for, and discussion of, any heterogeneity observed in the results of the review/ 15. Publication bias assessed/ 16. Include conflict of interest. *AMSTAR2 was used to critically appraise the reporting quality of each included SR. The overall confidence of each SR was graded as "high", "moderate", "low", "critically low".

References

1. Wang, Y.T.; Qi, Y.; Tang, F.Y.; Li, F.M.; Li, Q.H.; Xu, C.P.; Xie, G.P.; Sun, H.T. The effect of cupping therapy for low back pain: A meta-analysis based on existing randomized controlled trials. Journal of back and musculoskeletal rehabilita-tion 2017, 30, 1187-1195, doi:10.3233/bmr-169736.

2. Huang, C.Y.; Choong, M.Y.; Li, T.S. Effectiveness of cupping therapy for low back pain: a systematic review. Acupunc-ture in medicine : journal of the British Medical Acupuncture Society 2013, 31, 336-337, doi:10.1136/acupmed-2013-010385.

3. Azizkhani, M.; Ghorat, F.; Soroushzadeh, S.M.A.; Karimi, M.; Yekaninejad, S. The effect of cupping therapy on non-specific neck pain: a systematic review and meta-analysis. Iranian Red Crescent Medical Journal 2018, 20, doi:10.5812/ircmj.55039.

4. Cao, H.; Li, X.; Yan, X.; Wang, N.S.; Bensoussan, A.; Liu, J. Cupping therapy for acute and chronic pain management: A systematic review of randomized clinical trials. Journal of Traditional Chinese Medical Sciences 2014, 1, 49-61, doi:10.1016/j.jtcms.2014.11.003.

5. Kim, J.I.; Lee, M.S.; Lee, D.H.; Boddy, K.; Ernst, E. Cupping for treating pain: a systematic review. Evidence-based com-plementary and alternative medicine : eCAM 2011, 2011, 467014, doi:10.1093/ecam/nep035.

6. Kwon, Y.D.; Cho, H.J. Systematic review of cupping including bloodletting therapy for musculoskeletal diseases in Ko-rea. Korean J Orient Physiology Pathology 2007, 21, 789-793.

7. Wood, S.; Fryer, G.; Tan, L.L.F.; Cleary, C. Dry cupping for musculoskeletal pain and range of motion: A systematic review and meta-analysis. J Bodyw Mov Ther 2020, 24, 503-518, doi:10.1016/j.jbmt.2020.06.024.

8. Al Bedah, A.M.; Khalil, M.K.; Posadzki, P.; Sohaibani, I.; Aboushanab, T.S.; AlQaed, M.; Ali, G.I. Evaluation of wet cup-ping therapy: Systematic review of randomized clinical trials. Journal of alternative and complementary medicine (New York, N.Y.) 2016, 22, 768-777, doi:10.1089/acm.2016.0193.

9. Lee, M.S.; Choi, T.Y.; Shin, B.C.; Kim, J.I.; Nam, S.S. Cupping for hypertension: a systematic review. Clinical and exper-imental hypertension (New York, N.Y. : 1993) 2010, 32, 423-425, doi:10.3109/10641961003667955.

10. Li, M.; Zhao, Y.; Laing, X.; Hu, M.; Yan, X.; Zhao, L. Meta analysis of efficacy of combined with cupping therapy in psoriasis vulgaris. World Clinical Drugs 2020, 41, 711-718, 741.
